# Published correlational effect sizes in social and developmental psychology

**DOI:** 10.1098/rsos.220311

**Published:** 2022-12-21

**Authors:** Josefína Weinerová, Dénes Szűcs, John P. A. Ioannidis

**Affiliations:** ^1^ Department of Psychology, University of Cambridge, Cambridge, England; ^2^ Meta-Research Innovation Center at Stanford (METRICS), Stanford University, Stanford, CA, USA

**Keywords:** effect size, statistical power, sample size, correlation

## Abstract

The distribution of effect sizes may offer insights about the research done and reported in a scientific field. We have evaluated 12 412 manually collected correlation effect sizes (Sample 1) and 31 157 computer-extracted correlation effect sizes (Sample 2) published in journals focused on social or developmental psychology. Sample 1 consisted of 243 studies from six journals published in 2010 and 2019. Sample 2 consisted of 5012 papers published in 10 journals between 2010 and 2019. The 25th, 50th and 75th effect size percentiles were 0.08, 0.17 and 0.33, and 0.17, 0.31 and 0.52 in Samples 1 and 2, respectively. Sample 2 percentiles were probably larger because Sample 2 only included effect sizes from the text but not from tables. In text authors may have emphasized larger correlations. Large sample sizes were associated with smaller reported correlations. In Sample 1 about 70% of studies specified a directional hypothesis. In 2010 no papers had power calculations, while in 2019 14% of papers had power calculations. These data offer empirical insights into the distribution of reported correlations and may inform the interpretation of effect sizes. They also demonstrate the importance of computation of statistical power and highlight potential reporting bias.

## 1. Introduction

Calculating effect sizes allows researchers to characterize the magnitude and practical importance of their findings (i.e. substantive significance), to compare findings across studies and determine statistical power and required sample sizes. However, interpreting effect sizes is not necessarily straightforward. Cohen [1] proposed conventional benchmarks, according to which an effect size can be considered small, medium or large. While this approach is widely used, it lacks context: what can be considered small or large effect size may differ between different research fields. Hence, using universal effect size benchmarks across different fields and research areas may lead to the overestimation or underestimation of effect size magnitudes in specific fields [2,3]. Due to this problem, researchers proposed that a particular effect size should be compared with the specific distribution of published effect sizes [2–4]. Nevertheless, field-specific published effect size distributions may also misrepresent true effect sizes because published effect sizes may be highly exaggerated [5,6]. To compare the above approaches, here, we explored the distribution of published statistically significant and non-significant effect sizes and compared them with Cohen's [1] effect size benchmarks in the fields of social and developmental psychology. While some previous studies on the topic have included non-significant effect sizes, to the best of our knowledge none have compared statistically significant and non-significant effect sizes in their analyses. However, this is important as statistically non-significant effect sizes may be less exposed to effect size exaggeration. When compiling effect size distributions, we have also considered the interplay of degrees of freedom (in the case of correlation degrees of freedom = sample size minus 2) and published effect sizes as studies with larger degrees of freedom tend to publish smaller statistically significant effect sizes [6,7].

Table 1 shows results from studies in the psychological science and related areas evaluating effect size distributions in specific fields [2–4,8,9,11,12]. Apart from Hemphill [8] and Hartgerink *et al*. [10], all of these studies used the 25th, 50th and 75th percentiles as proxies for small, medium and large effect sizes. While Cohen did not suggest using these percentiles as benchmarks, their use is motivated by the definition of effect sizes put forward by Cohen [13]. He defined a medium effect ‘*likely to be visible to naked eye of a careful observer (It has since been noted in effect size surveys that it approximates the average size of observed effects in various fields)*’ [14, p. 156]. Small effect size was defined as noticeably smaller than medium effect size but non-trivial. Large effect size was defined as different from the medium effect size by the same degree but in the opposite direction [13].

**Table 1.** Summary of studies analysing effect size distribution across different fields and subfields in the psychological sciences and related areas. Note. If other types of effect sizes were collected, they were transformed to the type used for the analysis. NA = data not available. Type of effect size is specified in the 3rd column.

study	research area	type of effect	number of records/studies	25th percentile	50th percentile	75th percentile	sample size quartiles	comments
Hemphill [8]	psychological assessment	correlation	78 meta-analyses	NA	NA	NA		based on data from two reviews, the study suggests to categorize *r* < 0.20 as small, *r* from 0.20 to 0.30 as medium and *r* > 0.30 as large.
	psychological treatment		302 meta-analyses	NA	NA	NA		
Richard *et al*. [9]	social psychology	correlation	474/322 meta-analyses	NA	0.21	NA	NA	mean, not median reported, collected aggregated effect sizes from meta-analyses.
Gignac & Szodorai [4]	individual differences	correlation	708 /87 meta-analyses	0.11	0.19	0.29	NA	708 observed correlations, 345 true score correlations.
Quintana [3]	heart-rate variability	Cohen's *d*	297 /16 meta-analyses	0.26	0.51	0.88	NA	sample also includes non-significant correlations.
Hartgerink *et al*. [10]	psychology	eta	223 082 records	NA	NA	NA	NA	compared with Cohen's benchmarks, 7% of effect sizes were zero to small, 23% were small to medium, 27% were medium to large and 42% were large or larger
Rubio-Aparicio *et al*. [11]	treatment effectiveness, within-groups design	Cohen's *d*	NA/11 meta-analyses	0.64	0.75	1.26	16; 19.5; 37.5	use mean effect sizes for the distribution.
	treatment effectiveness, between-groups design	Cohen's *d*	NA/44 meta-analyses	0.25	0.41	0.69	32; 46.5; 64	
Schäfer & Schwarz [2]	without preregistration	correlation	900/900 empirical articles	0.20	0.36	0.62	NA; 89; NA	used only first effect size linked to key research question in each study.
	with preregistration	correlation	93/93 empirical articles	0.04	0.16	0.41	NA; 268; NA	
Lovakov & Agadullina [12]	social psychology	correlation	12 170/75 meta-analyses	0.12	0.24	0.41	NA	authors report weak negative relationship between effect size and sample size.
		Cohen's *d*	6447/59 meta-analyses	0.15	0.36	0.65	NA	

The percentile values from the above studies can be used as an empirical comparison against Cohen's benchmarks. For example, a recent analysis of 708 correlation coefficients collected from meta-analyses focusing on individual differences studies estimated the 25th, 50th and 75th percentiles of the effect size distribution at *r* = 0.11, *r* = 0.19 and *r* = 0.29, respectively [4]. These effect sizes are much lower than effects generally considered small, medium and large, respectively. Conversely, Quintana [3] looked at effect size estimates from meta-analyses of heart rate variability case control studies and found that Cohen's benchmarks slightly underestimate the effect size magnitudes in that area of research. Certainly, it should be acknowledged that the observed effect sizes may represent a combination of true effects and bias. Bias typically (but not necessarily always) will tend to make them bigger.

### 1.1. Limitations of studies on effect size distributions

To date, most studies focusing on the estimation of effect size distributions in different fields have used data from meta-analyses. This approach has the advantage of accessing effect sizes from a specific field or related to a specific question, but it is subject to limitations. First, meta-analyses are at risk of relying on exaggerated published effect sizes. Second, the decision to exclude or include certain papers represents a secondary source of researcher degrees of freedom in addition to the one present in the primary literature. Third, only a few relevant papers to date have incorporated statistically non-significant effect sizes in its sample [3,10]. However, including these effect sizes in analyses is important because non-significant results are probably less exposed to effect size inflation bias than statistically significant results. Fourth, while a few relevant studies commented on the relationship of sample sizes and statistical significance [2,3,12], to the best of our knowledge no studies focused on the interpretation of effect sizes have examined how effect size distribution will be affected by sample size distribution.

### 1.2. The current study

Here, we address four limitations of the literature. First, we collected a large amount of data from the primary literature including correlations, sample sizes and *p*-values. Second, considering sample sizes in analyses allowed us to determine how effect size distributions vary as a function of sample size. Third, we collected both statistically significant and non-significant effect sizes that allowed us to gain a more balanced impression about expected effect sizes. Fourth, to gain an impression of potentially changing research practices we also determined the temporal change in effect size and sample size distributions during the last decade.

We collected two samples of correlation coefficients from the social and developmental psychology literature. In Sample 1 we have manually extracted 12 412 records of statistical information including correlation coefficients (*r*), sample size (*N*) and *p*-values from 178 papers including 243 studies published in 2010 and 2019 in six major journals in social and developmental psychology. By records, we mean each individual occurrence of correlation coefficients fulfilling the inclusion criteria within the study. In Sample 2 we have used a computer algorithm to extract 31 157 statistical records: *r* values, degrees of freedom (d.f.) and *p*-values from all papers published in 10 social and developmental psychology journals (six of which have been used for Sample 1 data collection as well) between the years 2010 and 2019. The data enabled us to assess the distribution of published sample sizes and correlation effect sizes, compare them between two subfields of psychology and to understand how correlation distributions have changed during the past decade.

## 2. Methods

The study has been preregistered on the Open Science Framework at https://osf.io/u96yn/.

### 2.1. Sample 1

Sample 1 consisted of 12 412 correlation coefficients and related statistical information which were extracted from 243 studies published in 2019 and 2010. For the year 2019, we collected 6895 correlations from 127 studies. For the year 2010, we collected 5517 correlations from 116 studies. All records collected were included in the analysis.

In our preregistration, we had set out the aim to collect records from half a year's worth of issues for each journal in each year. This turned out to be unmanageable due to the number of potential records.

We manually extracted statistical information from social and developmental psychology papers published as pdf files in 2010 and 2019. Information on journals, papers and studies is shown in table 2. We sampled papers from three journals focused more on social psychology and three journals focused more on developmental psychology. The specialization of the journals was determined using the subject categories on SCImago Journal and Country Rank website [14]. We specifically chose those journals that focus primarily on empirical articles and have issues available for both the year 2019 and 2010. Additionally, the three journals for each subfield were selected so that their impact factors span different levels to keep the sample as representative of the field as possible.

**Table 2.** Data collected in Sample 1. Note. The table shows the names, 5-year impact factors and issues of journals used for Sample 1 data collection. In the papers/studies column, we see the number of papers (before slash) and the number of studies (after slash) collected from a given journal in either 2010 or 2019. Data were collected from all eligible papers contained in the mentioned issues. 5-year IF = 5-year impact factor; papers = number of collected separate scientific reports published as a pdf file; studies = number of experiments with separately defined sample, methods and results reported within the papers; records = number of statistical records collected from each journal (1 record = 1 correlation with associated data).

journal	5-year IF	2010			2019		
		volume/issue	papers/studies	records	volume/issue	papers/studies	records
developmental psychology:							
Child Development	6.151	81/2, 81/4	*N* = 16/18	*N* = 881	90/1, 90/5	*N* = 38/41	*N* = 2679
Journal of Child Psychology and Psychiatry	7.597	51/6, 51/8, 51/10	*N* = 17	*N* = 863	60/1, 60/3, 60/5	*N* = 8	*N* = 486
Developmental Psychology	4.798	46/2, 46/4	*N* = 23/25	*N* = 2082	55/1	*N* = 11/12	*N* = 1019
social psychology:							
Social Psychological and Personality Science	3.438	1/2, 1/3, 1/4	*N* = 15/20	*N* = 268	10/2	*N* = 11/21	*N* = 684
Journal of Personality and Social Psychology	7.293	98/3, 99/5	*N* = 11/27	*N* = 660	116/1, 116/2	*N* = 12/23	*N* = 977
European Journal of Personality	4.620	24/2, 24/4, 24/6	*N* = 9	*N* = 763	33/1	*N* = 7/22	*N* = 1050
total *N*			*N* = 91/116	*N* = 5517		*N* = 87/127	*N* = 6895

Issues from each journal were selected at random without previously reading any papers. Once we realized that collecting half a year worth of issues was unrealistic, we tried to balance the choice of issues over the whole year, but this was not always possible due to some data being already collected at this point. Data for year 2019 were collected prior to data for year 2010. Data from all eligible studies within these selected issues were extracted. If there were multiple studies reported within a paper, the data for each study were recorded separately.

Studies were included in the data if they reported correlation coefficients in their Results section or in the electronic supplementary material. The method for data extraction was developed on a sample of 58 studies from 33 papers (2923 records) published in the 2019 issues from all six journals in table 2. These papers were part of the final sample as well.

The pdf files were downloaded from the online platforms of each journal. The extracted data included: all correlation coefficients reported in Results sections or electronic supplementary material, in tables and text; reported significance levels or *p*-values corresponding to these correlation coefficients; the type of correlation coefficient used; whether the correlation was computed between two different constructs or between the same construct measured at two time points; whether correlation coefficients were reported in the Results sections or in electronic supplementary material and whether correlation coefficients were reported in correlation tables or in the text.

Additionally, we also extracted the following information for each study: journal name; first author of study; topic of the paper; overall sample size; use of power calculation to estimate sample size; whether alternative hypotheses predicted a directional effect, an effect in either direction, or a threshold value at which the effect would be considered large enough to provide evidence for the hypothesis; any comments on the null hypothesis or the null hypothesis set other than specifying the null hypothesis as *r* = 0 correlation. For the 2019 papers, we also recorded whether each study was preregistered (preregistration was not yet typically used in psychology in 2010).

When examining reported alternative hypotheses, we found that it was not possible to distinguish between hypotheses set *a priori* and *post hoc*. This caveat also applies to preregistered studies as it is possible that only certain correlations were included in the preregistration. When study preregistration was available, the hypotheses reported in the published studies and in the preregistrations were compared.

The median number of correlations extracted per study was 18 for the year 2010 and 34 for 2019. This increase in the median of the number of correlations published per study was mostly due to the Social Psychological and Personality Science journal (SPPS; increase by 200%), the Journal of Personality and Social Psychology (JPSP; increase by 150%) and the Journal of Child Psychology and Psychiatry (JCPP; increase by 94%). For the Developmental Psychology journal (DevPsy) the median number of correlations per study increased by 42% and for the Child Development journal (ChildDev) by 32%. For the European Journal of Personality (EJP) the number of correlations published in 2019 was 30% lower than the one for 2010.

### 2.2. Sample 2

In total 31 157 records were extracted for Sample 2. Of these records, 579 came from papers also included in Sample 1 (see details below).

Sample 2 was collected by an automated text mining algorithm adapted from Szűcs & Ioannidis [6]. We collected data from journal pdf files published in the same six journals as used in Sample 1 and the following four additional journals: Journal of Applied Developmental Psychology (5-year impact factor: 2.905), Journal of Experimental Child Psychology (5-year impact factor: 3.366), Journal of Experimental Social Psychology (5-year impact factor: 3.666) and Journal of Research in Personality (5-year impact factor: 3.365). All issues published between 2010 and 2019 were scanned for data. Table 3 summarizes the number of papers and the number of correlation records per paper by year, subfield and journal.

**Table 3.** The total number of records/papers extracted for different subfields and journals for the years 2010–2019. Note. ChildDev = Child Development, DevPsy = Developmental Psychology, JAppDevPsy = Journal of Applied Developmental Psychology, JCPP = Journal of Child Psychology and Psychiatry, JExpChildPsy = Journal of Experimental Child Psychology, EJP = European Journal of Personality, JExpSocPsy = Journal of Experimental Social Psychology, JPSP = Journal of Personality and Social Psychology, JResInPer = Journal of Research in Personality, SPPS = Social Psychological and Personality Science. The absence of *r* values in specific issues of journals is due to character encoding problems which have prevented our algorithm from extracting the data.

journal/year	2010	2011	2012	2013	2014	2015	2016	2017	2018	2019	total per journal/subfield
*developmental psychology*	*1124/226*	*1234/241*	*1154/233*	*1333/277*	*1621/310*	*1190/245*	*1349/280*	*1373/284*	*1567/309*	*1569/320*	*13 514/2725*
ChildDev	335/65	323/60	383/68	373/75	370/79	367/78	271/56	224/53	470/88	351/81	3467/703
DevPsy	263/52	229/64	251/65	317/71	474/106	237/46	274/62	391/88	472/84	314/74	3222/712
JAppDevPsy	110/29	87/16	49/16	84/17	208/26	77/18	94/22	117/34	129/23	247/44	1202/245
JCPP	262/53	323/46	205/44	197/43	202/37	213/49	232/48	241/41	175/41	155/36	2205/438
JExpChildPsy	154/27	272/55	266/40	362/71	367/62	296/54	478/92	400/68	321/73	502/85	3418/627
*social psychology*	*1295/219*	*1638/249*	*1764/280*	*1642/239*	*1457/219*	*1809/203*	*1631/182*	*1640/215*	*2215/239*	*2552/242*	*17 643/2287*
EJP	NA	15/1	193/29	257/28	244/20	257/24	253/30	158/19	155/20	497/25	2029/196
JExpSocPsy	285/72	285/90	319/90	320/75	347/78	262/56	139/37	299/71	538/79	394/71	3188/719
JPSP	527/84	752/91	683/99	517/67	451/77	953/83	775/67	623/63	711/72	660/73	6652/776
JResInPer	483/63	580/66	569/62	542/67	415/44	337/40	459/45	557/60	807/64	999/72	5748/583
SPPS	NA	6/1	NA	6/2	NA	NA	5/3	13/2	4/4	2/1	36/13
*total per year*	*2419/445*	*2872/490*	*2918/513*	*2975/516*	*3078/529*	*2999/448*	*2980/462*	*3013/499*	*3782/548*	*4121/562*	*Total N = 31 157/5012*

#### 2.2.1. Computerized extraction for Sample 2

The computer algorithm searched for specific word and symbol combinations for reporting *r* values, degrees of freedom and *p*-values. The algorithm searched the text of papers but not the tables for data records. In psychology, *r* values are often reported as ‘*r*(d.f.) = x.xx, *p* = y.yy’. The algorithm thus first identified whether pdf files included the symbol combination ‘*r* =’ or ‘*r*(d.f.)=’ neglecting spaces between the characters ‘*r*’, ‘(‘, ‘)’, ‘=’ and the string ‘d.f.’. If such combination was identified, the algorithm then extracted 56 characters starting with the ‘*r* =’.

Numerical values right after ‘*r* =’ and in the range of −1 to 1 were detected as *r* values. Values included in parentheses after ‘*r*’ (e.g. *r*(d.f.) =) were detected as degrees of freedom. Values reported after correlation values and preceded by ‘*p* =’, ‘*p*<’ or ‘*p*>’ were detected as *p*-values (irrespective of intervening spaces). The algorithm collected *r* values even in the case when degrees of freedom or *p*-values were not reported. The algorithm script is available at doi:10.5061/dryad.bg79cnpdw [15].

#### 2.2.2. Validation of the extraction procedure

Throughout the algorithm development phase, the efficiency of the text mining algorithm was validated by about a dozen separate checks on text mining outcomes. When errors were found, the text mining algorithm was perfected further to avoid the detected errors.

Error checks revealed that the algorithm has misidentified some negative correlations as positive. This happened due to very difficult-to-predict idiosyncratic changes in the character coding of pdf files. In order to avoid any sign errors we have only used absolute values in the analysis of Sample 2.

After the above initial checks to further validate the extraction algorithm the second phase of validation included drawing a sample of 20 random papers from the final data sample and manually verifying the accuracy of data extraction. The algorithm performed well. The randomly selected papers for validation included 392 data records in the text of papers. The algorithm has successfully identified 93% of them. The algorithm has missed 7% of *r* values and did not commit any false positive errors. The most common causes of missing records were line breaks within a correlation report and the use of subscript characters after the *r* value (e.g. ‘*r*_extraversion_ =’_)._ The list of studies used for validation can be found in the electronic supplementary material.

Additionally, during the data analysis we have found that the algorithm has not detected any data in some journals for some years. Upon checking these journals, we found this was due to character encoding issues within the pdf versions of the article. These issues do not allow for the possibility to even search for the symbol combination ‘*r* =’ using the search bar. However, we are interested in whole subfields rather than specific journals and this problem has not created an imbalance in the amount of data for each subfield.

### 2.3. Overlap between Sample 1 and Sample 2

**Figure 1.**
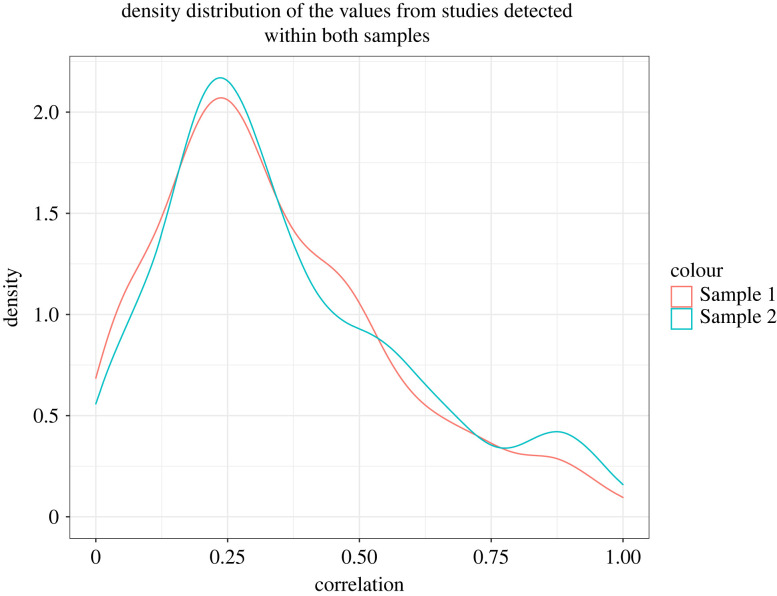
The density distribution of *r* values from overlapping papers in Sample 1 (*N* = 579) and Sample 2 (*N* = 669).

In Sample 1 there were 752 correlation values from 100 papers which were detected in text and could therefore theoretically be detected by the computerized extraction for Sample 2. Out of those, 28 papers with 173 values were not detected by the computerized extraction (e.g. because of special symbols used or because of verbal description of correlations, such as ‘correlation was 0.38’ instead of ‘*r* = 0.38’). This means that in total there was 72 papers and 579 correlation values which were included in both samples. The computerized extraction detected additional 90 correlation values in those shared papers. Considering that during validation the computer algorithm did not falsely detect any non-existent correlations, Sample 2 probably included the 90 additional records because Sample 1 records were collected only from the Results sections of the papers whereas the computerized extraction method collected all *r* values from all sections of papers. The distribution of correlation values from the overlapping studies is shown in figure 1.

Figure 1 shows overall high level of correspondence between the two samples. The 25th, 50th and 75th percentiles of *r* values in overlapping studies were 0.18, 0.29 and 0.47 for Sample 1 data and 0.19, 0.30 and 0.50 for Sample 2 data. This suggests that the additional correlations detected by the computerized extraction (*N* = 90) tended towards larger effect sizes (also consistent with the peak in density distribution between 0.75 and 1.00 for Sample 2 data in figure 1).

### 2.4. General data analysis

All analysis steps were performed in R programming software [16]. The code used for computation, analysis and visualizations can be found at https://osf.io/x45mj/. All analyses were done on absolute (unsigned) *r* values.

## 3. Results

### 3.1. Sample 1

#### 3.1.1. Distributions of *r* values and sample sizes

The number of *r* values reported in one study ranged between 1 and 441, with a median of 26 *r* values. Ninety-four per cent of the collected *r* values were reported in correlation tables. Studies with larger sample size reported more correlations in tables: studies with sample size less than 100 reported 1080 correlation values and 85% of these were presented in tables. By contrast, studies with sample size greater than or equal to 100 reported 10 606 records and 95.6% of these were presented in tables. This could have important implications for studies using computerized methods to extract statistical information. Our data suggest that computerized methods extracting only data from text will not detect the majority of correlational effect sizes presented within the papers.

**Figure 2.**
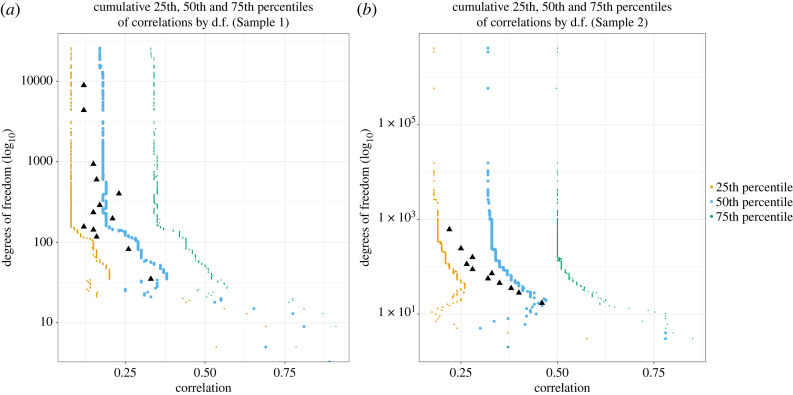
(*a*) The cumulative 25th, 50th and 75th percentiles of absolute *r* values by maximum degrees of freedom associated with correlation values for (*a*) Sample 1 (*N* = 12 406) and (*b*) Sample 2 (*N* = 3292). *r* values without corresponding degrees of freedom were excluded from this analysis. Degrees of freedom are plotted on log_10_ scale. The magnitude of 25th, 50th and 75th percentile decreases with increasing d.f. in both samples. The black triangles denote the median correlation for every 1000 values of Sample 1 and every 300 values in Sample 2 (we have used different amounts for this calculation because Sample 2 data have less available values for this analysis. The median of correlation values for groups within the data follows the same broad trend as the cumulative percentiles and decreases with the increase in degrees of freedom.

To better understand the interaction between sample size and *r* value magnitude we have mapped the cumulative 25th, 50th and 75th percentiles of correlation values by the maximum degrees of freedom associated with correlation values. Figure 2*a* shows that the magnitude of the percentiles decreased with including larger and larger degrees of freedom. As the percentiles for the larger degrees of freedom also include all *r* values with smaller degrees of freedom this suggests that with increasing degrees of freedom, overall sample *r* values are becoming smaller. Note that the percentile values were not weighted by sample size. That is, even without giving larger weight to larger studies as typically done in meta-analyses there is a substantial decrease in *r* values due to larger studies reporting much smaller effect sizes than smaller studies. Figure 2*b* confirms this trend also for data from Sample 2.

**Figure 3.**
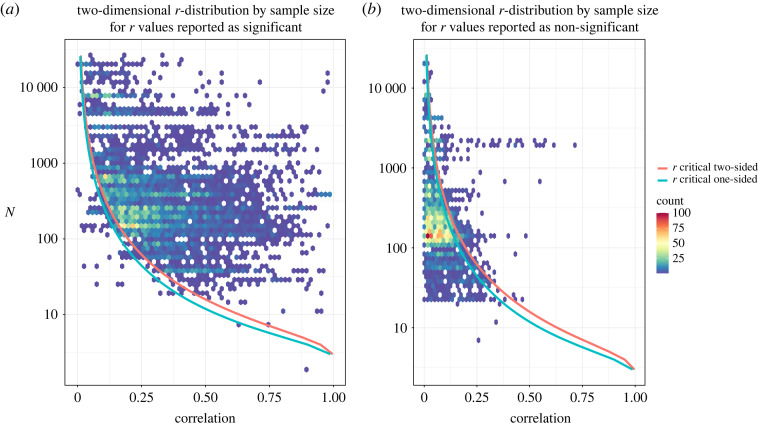
Bivariate distribution of sample sizes and *r* values for records reported as (*a*) statistically significant and (*b*) non-significant. The count of observations is colour coded. Sample sizes are plotted on a log_10_-spaced scale. The red line denotes the significance threshold *p* ≤ *α*, where *α* = 0.05 for two-sided test. The blue line denotes the significance threshold *p* ≤ *α*, where *α* = 0.05 for one-sided test.

We were interested in seeing how the distribution of reportedly significant and non-significant *r* values compared with the significance boundary. To this end, figure 3*a*,*b* shows the bivariate distribution of sample sizes and *r* values, for records reported as statistically significant and non-significant, respectively. The 25th, 50th and 75th percentile for all (statistically significant and non-significant) 12 412 correlations was 0.08, 0.17 and 0.33. The median sample size was 230, the 25th and 75th percentiles were 140 and 564 respectively. The density of statistically significant *r* values was highest near the significance boundary. There is also high concentration of non-significant *r* values with r < 0.1 between 100 < *N* < 300.

Figure 3*a,b* also indicates that some reportedly significant and non-significant *r* values cross the significance boundary. There are only few reported correlations which are too small to be statistically significant (figure 3*a*), and this may represent misreporting of significance status or statistical information. There are more reported correlations which are too large to be non-significant (figure 3*b*); in these cases, misreporting could also be at play, but it is also possible that some adjustment for multiplicity has been performed by the authors.

A total of 7163 (58%) correlations were reported as statistically significant, and 4825 (39%) correlations were reported as non-significant. The remaining 3% (424 correlations) were reported without specified significance. A total of 144 correlations were reported as *r* = 0.

**Figure 4.**
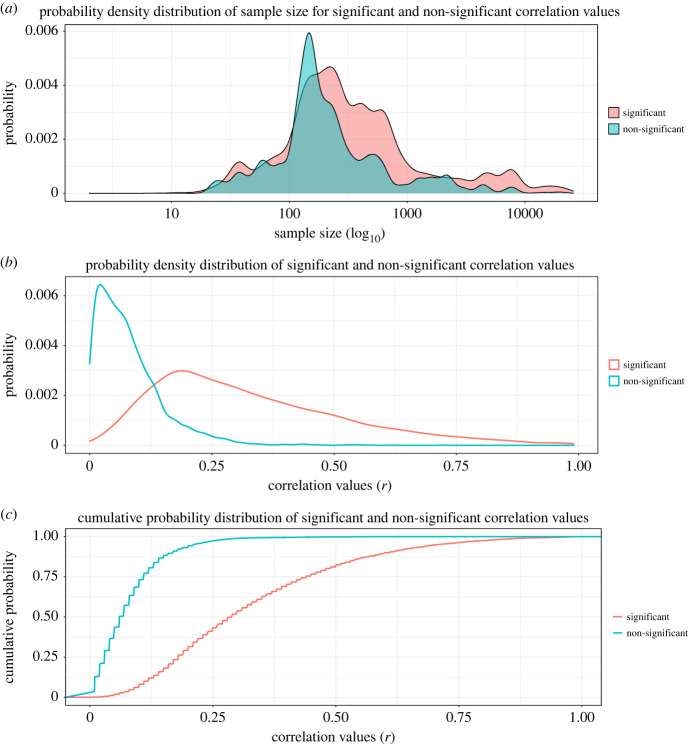
(*a*) The univariate density distribution of sample sizes separately depending on whether the *r* value associated with given sample size was reported as significant (*N* = 7163) or non-significant (*N* = 4825). While the two distributions are largely overlapping with smaller sample size values, the prevalence of significant values increases at larger sample sizes. (*b*) The univariate density distribution of correlation values depending on their significance. (*c*) The cumulative probability distribution of correlation values depending on their significance.

Figure 4*a* shows the probability density of sample sizes for statistically significant and non-significant *r* values. Figure 4*b*,*c* shows the probability density and cumulative density of correlation values, respectively. As expected, with increasing sample size, the proportion of significant *r* values increases. This is because with larger sample size, smaller effect size will be detected as significant. For significant correlations, the 25th, 50th and 75th *r* value percentiles were 0.18, 0.29 and 0.44. For non-significant correlations, these percentiles were 0.03, 0.06 and 0.11, respectively.

In Sample 1, 752 records were collected from text (6%). Out of those 119 records had no specified significance status. We were interested in whether authors do report significant correlations more in text rather than tables. Our results would indicate that this is correct, as about 83% of correlations in the text were statistically significant, whereas only 59% of correlations in tables were statistically significant. However, the overall percentage of in-text correlations is very low in Sample 1, which makes this result tentative.

The 25th, 50th and 75th percentiles of *r* values were 0.16, 0.28 and 0.46 for those reported in text and 0.07, 0.17 and 0.32 for those reported in tables. Table 4 shows the 25th, 50th and 75th percentiles for those correlations reported as significant and non-significant in text and tables.

**Table 4.** The proportions and 25th, 50th and 75th percentiles for correlations reported in text and in tables depending on the reported significance.

reported in:	number of correlations	percentiles		
		25th	50th	75th
text significant	511	0.22	0.33	0.51
text non-significant	122	0.03	0.09	0.17
tables significant	6652	0.18	0.28	0.43
tables non-significant	4703	0.03	0.06	0.11

We also wanted to compare the distribution of *r* values and sample sizes across different years and subfields of psychology. Table 5 shows *r* value quartiles for different years and for different research areas. We calculated the exact *p*-values for those *r* values which were reported without a *p*-value or *α* (*N* = 424) and found that 384 *r* values were significant at *α* = 0.05 assuming a two-sided test. 396 *r* values were significant at *α* = 0.05 assuming a one-sided test. Note that assuming a one-sided test increases power, therefore smaller effect sizes will be detected as statistically significant.

**Table 5.** The 25th, 50th, and 75th percentiles of *r* values for subsets of data for the years 2010 and 2019 and for journals falling under social and developmental psychology. Note. *r*-value quartiles are shown separately for correlations reported as significant and non-significant. In developmental psychology 4558 (57%) records were reported as statistically significant, 3110 (39%) records were reported as non-significant, and 342 (4%) records were reported with no *p*-value. In social psychology 2605 (59%) records were reported as significant, 1715 (39%) as non-significant and 82 (2%) with no *p*-value.

		2010 *N* = 5517	2019 *N* = 6895	developmental *N* = 8010	social *N* = 4402
sample size		111; 147; 330	195; 267; 913	119; 230; 527	145; 213; 560
all *r* values *N* = 12 412		0.08; 0.18; 0.33	0.07; 0.17; 0.34	0.07; 0.17; 0.33	0.08; 0.18; 0.33
*r* values reported as significant *N* = 7163		0.19; 0.29; 0.45	0.18; 0.28; 0.44	0.18; 0.29; 0.45	0.18; 0.28; 0.43
*r* values reported as non-significant *N* = 4825		0.034; 0.07; 0.12	0.03; 0.06; 0.10	0.03; 0.07; 0.11	0.03; 0.06; 0.10
developmental	*r* values	0.08; 0.18; 0.34	0.07; 0.16; 0.32		
	sample size	85; 147; 330	178; 267; 913		
social	*r* values	0.08; 0.17; 0.30	0.08; 0.18; 0.35		
	sample size	139; 145; 454	199; 300; 735		

**Figure 5.**
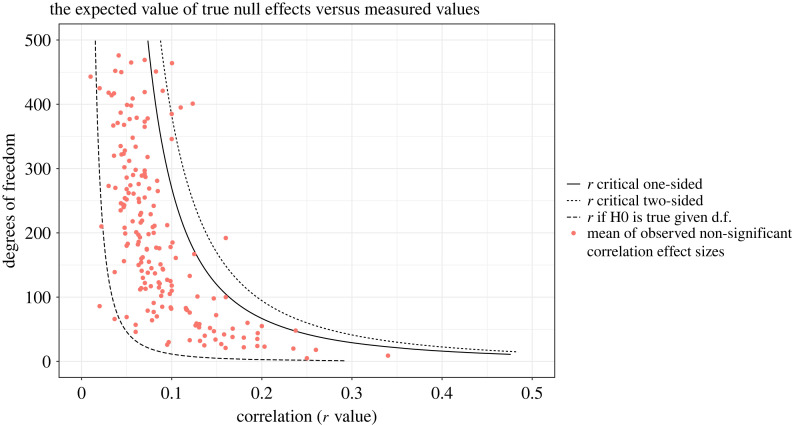
The expected and measured mean values of non-significant correlation effect sizes compared with the significance thresholds for two-sided (dotted line) and one-sided (solid line) test. Expected mean of non-significant correlation effect sizes if the null hypothesis was true (leftmost dashed line) was calculated as an integral of the critical threshold and 0, given particular degrees of freedom.

Figure 5 shows the expected and measured values of non-significant correlations. The disparity between the observed and expected values of non-significant effect sizes could be caused by the fact that non-significant results are a mixture of some results that arise from studies that target nil-null effects and some other studies that target non-null effects but end up being non-significant, or by the tendency to preferentially report ‘just non-significant’ values within the studies' results.

#### 3.1.2. Differences between years

In total there were 5517 *r* values for the year 2010 and 6895 for the year 2019. Table 5 summarizes the 25th, 50th and 75th percentiles of *r* values and sample sizes. There is a notable increase in the median sample size from 2010 to 2019. Conversely, *r* values remain remarkably similar. This is curious, as with increasing sample size, the effect size which will be detected as significant decreases.

#### 3.1.3. Differences between subfields

Table 5 also summarizes the 25th, 50th and 75th percentiles for *r* values and sample sizes for the subfields of social and developmental psychology. Sample sizes increased for both developmental (median: 147 to 267) and social psychology (median: 145 to 300) from 2010 to 2019. Conversely, *r* values were similar between the two fields, and they also did not change over time.

#### 3.1.4. Specification of study hypotheses

Studies were coded as having a directional hypothesis if at least one hypothesis specified the sign of an effect. Out of the 243 studies, 176 (72%) contained at least one hypothesis where the sign of the effect was specified; 67 of the studies (28%) had hypotheses which did not specify the expected sign of the effects. In 2010 there were 79 studies (68%) specifying the sign of an expected effect and 37 (32%) without specifying the sign. In 2019, there were 97 studies (76%) with directional hypothesis and 30 (24%) without a directional hypothesis. When using nil-null hypothesis and assuming high enough sample size, directional hypotheses have 50% chance of being found significant and non-directional hypothesis literally 100% chance. We have, however, not collected data on how the given hypotheses were actually tested.

Out of the 127 studies published in 2019, 18 (14%) included a power analysis. The expected effect size was reported in 13 of those. The rest did not specify the effect size, or the power calculation was done *a posteriori* on the detected effect. No power calculations were reported in studies published in 2010.

#### 3.1.5. Preregistrations

Seven studies in Sample 1 (4%) contained a link to a preregistration document. These studies included 329 correlations and came from five papers (three studies were part of one paper). The studies were published in the European Journal of Personality (five studies) and Social Psychological and Personality Science (two studies). Studies were preregistered on the Open Science Framework^1^ (six studies) or the AsPredicted platform^2^ (one study).

In two cases there was an extra hypothesis in the preregistration not stated in the published paper. In one case no hypotheses were mentioned in the preregistration. In one case the preregistered hypotheses were more precise (directional as opposed to explorative) than those stated in the study. In the four remaining studies, hypotheses were the same in both the preregistration and in the published paper. The 25th, 50th and 75th percentiles for correlations collected from preregistered studies were *r* = 0.07, 0.18 and 0.35 and *N* = 157, 213 and 264.

### 3.2. Sample 2

#### 3.2.1. Distributions of *r* values and degrees of freedom

**Figure 6.**
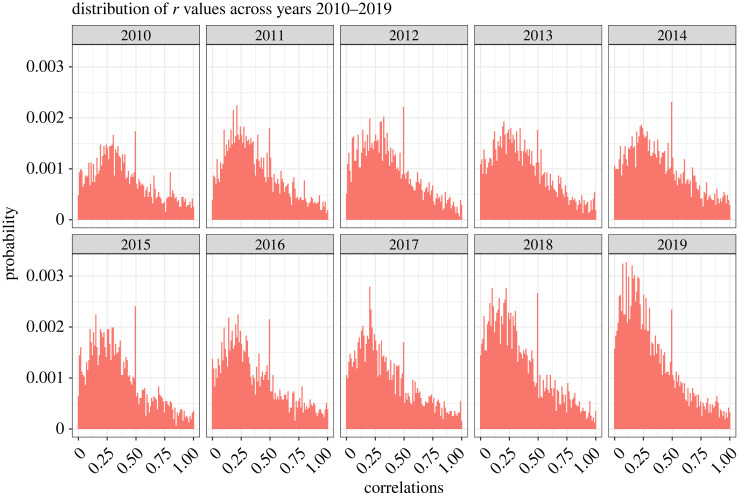
Probability distribution of all *r* values in Sample 2 per year.

**Figure 7.**
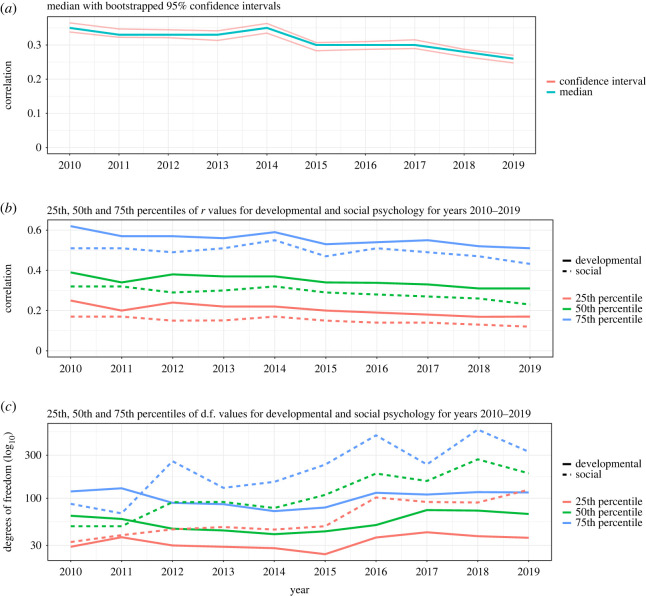
(*a*) Medians of all Sample 2 data across years with 95% bootstrap confidence intervals. The bootstrap method used is ‘standard normal interval’ and computed in R as part of the boot package [17]. *N* of simulations = 10 000. (*b*) The 25th, 50th and 75th percentiles of *r* values for developmental (*N* = 13 514) and social (*N* = 17 643) psychology. (*c*) The 25th, 50th and 75th percentiles of all collected degrees of freedom for developmental (*N* = 1813) and social (*N* = 1479) psychology. Degrees of freedom for social psychology have increased across the years while they have stayed about the same for developmental psychology. Degrees of freedom are plotted on a log_10_-spaced scale.

A total of 31 157 correlations were extracted for the years 2010–2019. The 25th, 50th and 75th percentiles for all *r* values were 0.17, 0.31 and 0.52. Figure 6 shows the distribution of *r* values in each year. Figure 7*a* shows the median *r* values with bootstrapped 95% confidence intervals. The median *r* value decreased from 0.35 in 2010 to 0.26 in 2019. Note that this is in contrast with the results in Sample 1 where the correlation values remained similar between the years. Figure 7*b*,*c* shows the 25th, 50th and 75th percentiles for *r* values and degrees of freedom across the years for different subfields. Degrees of freedom were reported with 3292 *r* values (11%). The 25th, 50th and 75th percentiles for the degrees of freedom were 37, 72 and 144. Degrees of freedom increased from a median of 53 in 2010 to a median of 114.5 in 2019 which was also true in Sample 1. As an exception from this trend, there was a decrease in degree of freedom from the year 2018 (median = 184) to 2019 (median = 114.5).

Table 6 shows the number of records with degrees of freedom for each year and the correlation between degrees of freedom and *r* value magnitude. In early years there was a negative correlation between the degrees of freedom and *r* value magnitude, but this correlation gradually disappeared by 2019.

**Table 6.** The number of records including degrees of freedom per year and the Spearman's rho correlations of absolute *r* values and degrees of freedom for each year. Note. Spearman's rho correlation was used after the Shapiro–Wilk normality test has shown that the data is not normally distributed.

year	2010	2011	2012	2013	2014	2015	2016	2017	2018	2019
number of records	247	297	405	399	441	320	289	299	331	264
Spearman's rho correlation between the *r* values and sample size with 95% confidence interval	−0.31 (−0.43; −0.18)	−0.22 (−0.33; −0.10)	−0.32 (−0.42; −0.23)	−0.07 (−0.17; 0.04)	−0.29 (−0.38; −0.20)	−0.19 (−0.30; −0.08)	−0.24 (−0.36; −0.12)	−0.09 (−0.21; 0.03)	−0.05 (−0.16; 0.06)	−0.01 (−0.14; 0.12)

#### 3.2.2. Differences between subfields

The comparison of subfields of psychology in Sample 2 yielded the following results. The 25th, 50th and 75th percentiles for all *r* values collected from journals focused on developmental psychology were 0.20, 0.35 and 0.56 (*N* = 13 514). In the case of journals focused on social psychology the respective *r* values were 0.15, 0.28 and 0.49 (*N* = 17 643). The overall percentiles of degrees of freedom were 20, 53 and 97 (*N* = 1813) for developmental psychology and 51, 103 and 237 (*N* = 1479) for social psychology. The sample sizes of degree of freedom values are smaller than the sample sizes of correlation values because degrees of freedom could be extracted only for a subset of the *r* values in Sample 2.

The 25th, 50th and 75th percentiles of *r* values and degrees of freedom for the two subfields across the years are shown in table 7. The magnitudes of degrees of freedom have increased across the years while *r* value magnitudes have decreased for both fields.

**Table 7.** The 25th, 50th and 75th percentiles for each year divided into *r* values and degrees of freedom published in journals specializing in developmental psychology and journals specializing in social psychology. Note. *r* = *r* values; d.f. = degrees of freedom. For both subfields the median *r* value for 2019 is slightly lower than the median *r* value for 2010. For both subfields the median d.f. for 2019 is higher than the median d.f. for 2010. There is no clear decreasing pattern across years for either subfield. The number of records collected for each group is shown.

subfield year	developmental psychology		social psychology	
	*r*	d.f.	*r*	d.f.
2010	0.25; 0.39; 0.52 (*N* = 1124)	29; 64; 119 (*N* = 125)	0.17; 0.32; 0.51 (*N* = 1295)	33; 49; 87 (*N* = 122)
2011	0.20; 0.34; 0.57 (*N* = 1234)	37; 59; 129 (*N* = 132)	0.17; 0.32; 0.51 (*N* = 1638)	39; 49; 68 (*N* = 165)
2012	0.24; 0.38; 0.57 (*N* = 1154)	30; 46; 89 (*N* = 237)	0.15; 0.29; 0.49 (*N* = 1764)	45; 91; 258 (*N* = 168)
2013	0.22; 0.37; 0.56 (*N* = 1333)	29; 44; 86 (*N* = 288)	0.151; 0.300; 0.510 (*N* = 1642)	48; 91; 131 (*N* = 111)
2014	0.22; 0.37; 0.59 (*N* = 1621)	28; 40; 72 (*N* = 305)	0.17; 0.32; 0.55 (*N* = 1457)	45; 78; 152 (*N* = 136)
2015	0.20; 0.34; 0.53 (*N* = 1190)	24; 43; 79 (*N* = 136)	0.15; 0.29; 0.47 (*N* = 1809)	49; 109; 236 (*N* = 184)
2016	0.19; 0.34; 0.54 (*N* = 1349)	37; 51; 115 (*N* = 180)	0.14; 0.28; 0.51 (*N* = 1631)	102; 188; 498 (*N* = 109)
2017	0.18; 0.33; 0.55 (*N* = 1373)	42; 74; 110 (*N* = 173)	0.14; 0.27; 0.49 (*N* = 1640)	91; 156; 237 (*N* = 126)
2018	0.17; 0.31; 0.52 (*N* = 1567)	38; 73; 117 (*N* = 91)	0.13; 0.26; 0.47 (*N* = 2215)	90; 270; 577 (*N* = 240)
2019	0.17; 0.31; 0.51 (*N* = 1569)	37; 67; 116 (*N* = 146)	0.12; 0.23; 0.43 (*N* = 2552)	124; 189; 328 (*N* = 118)

## 4. Discussion

In this study, we have determined the distribution of correlation coefficients in five developmental and five social psychology journals. We used two samples. Sample 1 included correlations from both text and tables. Sample 2 included correlations only from the text. Sample 2 correlations were larger than those of Sample 1, probably because the correlations chosen to be presented in the text were a biased subsample of all correlations calculated. Hence, larger correlations may have been reported in the text than in tables. In Sample 2 the magnitude of correlations decreased over the decade we examined, whereas they remained stable in Sample 1.

### 4.1. Implications for power calculations

In Sample 1 the 25th, 50th and 75th percentiles of all correlation effect sizes were *r* = 0.08, 0.17 and 0.33, much smaller than Cohen's estimates for small, medium and large effect sizes (*r* = 0.1, 0.3 and 0.5, respectively). Considering only correlations clearly reported as statistically significant the 25th, 50th and 75th percentiles were 0.18, 0.29 and 0.44, reasonably close to Cohen's [1] estimates. However, considering only correlations reported as statistically non-significant, the respective percentiles were very small (0.03, 0.06 and 0.11). This is to be expected as larger magnitudes of effect sizes would by definition be detected as statistically significant.

If we consider the effect sizes from both statistically significant and non-significant reports to be the best overall effect size estimates, the implications for power calculations are clearly profound: the relationship between power and effect size is not linear, even a seemingly small decrease in effect size may translate to a considerable change in sample size. For example, relying on Cohen's benchmarks would suggest that a sample size of 84 is required to detect a ‘medium-sized’ effect of *r* = 0.3. By contrast, our data suggest that a 219% larger sample size of 268 would be necessary (*r* = 0.17, power = 0.8, *α* = 0.05, two-sided test).

It is a question whether field-wide estimates of effect size distribution such as those presented here are the right basis for power calculation, or whether one should focus on the distribution of effect sizes from previous studies looking at similar problems. The latter approach would probably be more precise as long as studies looking at the specific question of interest are well-powered and unbiased. One has to question in each case whether the available results are less biased in the studies targeting similar questions or in the larger discipline. Discipline-wide effect size distributions can also be interesting in order to determine the overall distribution of effects one can expect within a wider field. Here we have focused on psychological sciences, but similar considerations may apply also to other scientific fields, e.g. empirical distributions of effect sizes have been studied also in medical disciplines [18–20].

### 4.2. Differences between samples

There was a pronounced difference in the percentiles of the two samples we collected. In Sample 2 the 25th, 50th and 75th percentiles were 0.1–0.2 larger than in Sample 1: 0.17, 0.31 and 0.52. This difference can be attributed to the fact that the automatic extraction algorithm used to collect Sample 2 was able to extract only correlations reported in the text. By contrast, in Sample 1 only 6% of all records were collected from text. Indeed, considering only Sample 1, we found that correlation effect sizes were about 0.1–0.2 larger in the text than in tables. This disparity may arise because correlation tables often include correlations between all observed variables whereas correlations mentioned in the text are more likely to be interpreted by the narrative of papers, so they are more likely to be related to variables in the focus of the study, or how the study had been written up. Text bodies may also contain larger correlations, because they draw more attention and are considered worth mentioning in the text, regardless of whether they reflect primary analyses or secondary, exploratory ones. Alternatively, the disparity could also be caused by the mean degrees of freedom being smaller in Sample 2 than in Sample 1. Given that in Sample 1 we can see higher proportion of significant correlations in text, the former explanation seems to play at least a partial role. In any case, this points to an important problem for researchers deciding between manual and computerized collection of correlational effect sizes to study their distribution, as computerized extraction methods usually sample only information from text.

### 4.3. Temporal developments in effect size and sample size distributions

The recent awareness of a high number of false positive findings in published literature [21,22] has led to calls to increase sample sizes in various research areas [6,23–25]. In our manually collected Sample 1 data, we found a median sample size increase in both developmental (from 147 to 267) and social psychology (145 to 300). Effect sizes remained stable in both subfields (0.17–0.18 and 0.18–0.16 in developmental and social psychology respectively). By contrast, in our Sample 2 data we found that median degrees of freedom increased in both fields but more modestly so, especially in developmental psychology. Between 2010–2012 and 2017–2019 effect sizes declined in both fields: 0.36 to 0.30 in developmental, and 0.29 to 0.23 in social psychology (Sample 2).

The correlation effect sizes in Sample 2 were negatively correlated with associated sample size in the earliest publication years we studied. However, this negative correlation gradually disappeared by 2019. This may be explained by less selective reporting of correlations in text in recent years.

### 4.4. Correlation distributions differ between subfields of psychology

Multiple authors pointed out that using universal effect size benchmarks may lead to the underestimation or overestimation of the effect sizes in research subfields [2–4]. Here, we found that the 25th, 50th and 75th *r* value percentiles were very similar in developmental and social psychology studies in Sample 1 (0.07, 0.17, 0.33, and 0.08, 0.18, 0.33, respectively). In Sample 2 there was more pronounced difference in *r* value percentiles between the two fields (0.20, 0.35 and 0.56, and 0.15, 0.28 and 0.49 for developmental and social psychology respectively). If Sample 2 picked up correlations more likely to be the foci of the studies (reported in the text), this would suggest that while the distribution of reported correlations in the two fields is very similar, the focus of the two fields is on correlations of different magnitudes. Alternatively, researchers in developmental psychology may prefer to highlight larger correlations.

The between-field comparison of *r* values and degrees of freedom was hindered by the fact that degrees of freedom could be extracted only for a subset of values in Sample 2, as most often they were not reported with each *r* value.

### 4.5. Sample size and effect sizes

When relying on null-hypothesis significance testing, having larger sample sizes allows one to detect smaller effect sizes as statistically significant. In fields where low sample sizes are typical this will lead to exaggeration of effect sizes in the published literature because studies with low sample sizes can only produce statistically significant results if effects are relatively large [26]. However, due to sampling variability large effects sizes will be detected from time to time even if the true effects tested are small or null [6]. This argument is supported by findings from large-scale replication efforts. The Open Science Collaboration [27] study conducted replication of 100 psychology studies and found that the mean effect size of the replications (*r* = 0.197) was roughly half the magnitude of the mean effect size published in the original studies (*r* = 0.403). Furthermore, a recent report focused on emotion research showed that effect sizes reported in highly cited observational and experimental studies are on average about twice larger compared with the largest sample studies on the same topic [7].

Hence, published field-specific effect size distributions probably depend on study sample sizes. Our data showed clear evidence of this expectation: We found that as records with larger and larger sample sizes were considered, smaller and smaller median *r* values were found.

Schäfer & Schwarz [2] have suggested an alternative explanation for the above potential regularity arguing that the negative relationship between sample size and effect size may also arise because in research fields with large effects researchers have learned that small sample size is sufficient while in fields with small effects researchers would aim for larger sample sizes. However, this suggestion cannot explain the pattern of our data: here, we observed a strong decline in the magnitude of cumulative percentiles of *r* values with increasing sample sizes even within the same research field and even while the decrease in *r* value magnitudes across years was relatively small. Hence, it is likely that published correlation magnitudes are driven by study sample sizes simply because larger studies can publish more statistically significant small effects. This suggests that researchers cannot simply determine ‘true’ expected effect sizes by looking at some published papers. Rather, the decline of effect sizes with increasing study sample size must be considered when trying to determine expected effect sizes.

### 4.6. Power calculations

Only 14% of the Sample 1 studies published in 2019 included a statistical power calculation (there were no statistical power calculations in studies published in 2010). This is more than twice the number reported by Szűcs and Ioannidis [24] for studies published in neuroimaging journals in 2017 (6.9% out of 130 studies) and 2018 (6.4% out of 140 studies). This may indicate either increased focus on statistical power calculations with time or may indicate that power calculations are more frequent in social and developmental psychology than in neuroimaging papers.

### 4.7. Preregistrations

Only seven studies in Sample 1 (4%) contained a link to a preregistration document (these studies included a total of 329 correlations). Schäfer & Schwarz [2] found that the median *r* value for preregistered studies was 55% lower than the median value for non-preregistered studies. However, in our sample, the 50th, and 75th *r* value percentiles for preregistered studies were slightly higher than those for all Sample 1, and the 25th percentile was slightly smaller. However, as our sample only included very few preregistered studies, our findings may not adequately represent effect and sample size differences between preregistered versus non preregistered studies.

It is noteworthy that very few studies in the sample were preregistered and that they have come from only two journals. This suggests that preregistration in 2019 was not yet widespread practice in many impactful journals in social and developmental psychology. Those studies that were preregistered have mainly used the Open Science Framework^3^

### 4.8. Hypothesis precision

Multiple authors pointed out that most hypotheses in psychological science tend to be directional at best [28–31]. Our data suggest that the situation has remained unchanged since the 1960s. No studies specified the looked-for effect size. Twenty-eight per cent of Sample 1 studies only specified hypotheses predicting an association but not the direction of the association; 72% of Sample 1 studies contained at least one hypothesis which specified a predicted direction of an effect; 8% more studies had directional hypotheses in 2019 than in 2010.

Specifying only the sign (direction) of an effect has been shown to lead to many false positives [32]. In studies with very high statistical power there is 50% probability of rejecting the null hypotheses. When not even the direction of an effect is specified, such studies can reject the null hypotheses with near certainty [28,30]. If the study hypotheses were specified after the results were known (HARKing; [33]), all studies of any sample size would be almost certain to detect a significant effect. It is also noteworthy that if studies do not specify an effect size sought then principled statistical power calculation becomes impossible.

### 4.9. Ambient correlational noise

Most psychological variables tend to correlate with each other simply because they are affected by the interactions of many background factors [30]. Hence, any randomly selected variables are likely to be at least remotely connected through a background network of interacting variables [31,34], leading to shared variance. Consequently, a randomly chosen pair of variables is likely to have non-zero absolute correlation [30,35,36]. This phenomenon is termed the ‘ambient correlational noise’ [30] or the ‘crud factor’ [37]. In our data the middle 95% of the overall correlation distribution ranged between 0 and 0.64 for Sample 1. The middle 95% of the distribution of non-significant correlations was between 0 and 0.21. That is, most absolute correlation values clearly departed from zero. It is also of note that the means of observed statistically non-significant correlations were larger than the means expected if the nil-null hypothesis was true. This could be because the nil-null hypothesis is correct only in a subset of the studies and therefore nearly all studies would measure larger than zero effect sizes. Nevertheless, this could also reflect the ambient correlational noise within the data.

These points are very important to consider when ‘real’ (not ‘statistical’) significance of results is evaluated: published studies often interpret statistically significant effect sizes in the 0.1 ≤ *r* ≤ 0.2 range. If the sample size is large enough, departure from zero will also be detected as statistically significant (and confidence intervals will exclude zero). However, since many correlations are likely to be small, correlation effect sizes of this magnitude may just reflect ambient correlational noise.

Given the problem of unspecified effect size magnitudes in hypotheses, there is a chance that many statistically significant results may arise due to the ambient correlational noise rather than due to the hypothesized associations. While sample size increase is usually viewed as an increase in the quality of the study, it is important to keep in mind that it should be accompanied by corresponding changes to the design of the study and optimally by a principled argument about the expected effect size and most recently, by preregistration. Otherwise, increased sample size may just lead to an increased number of false positive results [30].

## 5. Limitations

The automatic extraction algorithm used for Sample 2 could extract only correlations from the text but not from tables. The manually extracted data allowed us to determine that correlations reported in text were much less common than correlations reported in tables. However, we could not determine the ratio of correlations reported in tables to those reported in the text in Sample 2. Further, for analyses involving degrees of freedom and *p*-values in Sample 2, only a subset of the data could be used because these values were not reported with most *r* values.

While the validation procedure for Sample 2 has offered satisfactory results, it focused on checking papers from which there was at least one correlation successfully extracted. Therefore, there is a possibility that some papers containing correlations may not have been included in the sample if they only presented correlations in tables. However, when we compared the density distribution of correlation values from studies which were part of both samples, we found excellent correspondence.

Since we have collected all *r* values within the Results section (Sample 1) or within the text of the paper (Sample 2), some of these *r* values probably targeted the same or similar questions and are calculated on the same or similar sample, meaning that many or all of the *r* values collected within one paper are likely to be inherently correlated. Additionally, the researcher biases within the methods and analysis are likely to be the same for *r* values collected from the same papers. Given that we have shown that larger studies report smaller *r* values, this could skew the overall distribution of *r* values if either smaller studies or larger studies consistently reported larger number of *r* values. However, there was only weak correlation between the overall study sample size and number of *r* values reported (*r* = 0.13).

Finally, for the purpose of this study the two subfields presented have been defined by overall specialization of the journals considered in sampling.

## 6. Conclusion

As expected, we found that effect size distributions strongly depended on sample sizes: the larger the maximum sample size the smaller the corresponding effect size distribution quartiles. This suggests that effect sizes cannot be considered to be fixed and independent from sample sizes. Rather, larger studies will measure smaller effect size distributions than smaller studies. This observation has major implications for power calculations: many small and probably underpowered studies will report larger effect size quartiles than larger, well-powered studies. If power calculations are then based on effect sizes from small studies, future studies will also be small and underpowered and will also report relatively large effect sizes. Our observation also suggests that without considering how sample size affects effect size distributions, it cannot be determined whether large effects arise due to effect size inflation in small studies or whether they can really be expected in a field. Similarly, non-significant effect sizes should also be considered in effect size distributions because they may represent effects that are too small to be detected by underpowered studies.

## Data Availability

All data and code are available from the Dryad Digital Repository: https://doi.org/10.5061/dryad.bg79cnpdw [15]. The protocol and analysis plans were preregistered via OSF and can be accessed at https://osf.io/u96yn/. Supplementary material is available online [38].

## Footnotes

1
https://osf.io/

2
https://aspredicted.org/

3
https://osf.io/
